# Oxidation Resistance of Materials Based on Ti_3_AlC_2_ Nanolaminate at 600 °C in Air

**DOI:** 10.1186/s11671-016-1571-x

**Published:** 2016-08-09

**Authors:** Andrij Ivasyshyn, Orest Ostash, Tatiana Prikhna, Viktoriya Podhurska, Tatiana Basyuk

**Affiliations:** 1 H. V. Karpenko Physico-Mechanical Institute of the National Academy of Sciences of Ukraine, 5 Naukova Str., Lviv, 79060 Ukraine; 2 V. N. Bakul Institute for Superhard Materials of the National Academy of Sciences of Ukraine, Avtozavodskaya Str., 2, Kiev, 04074 Ukraine

**Keywords:** Nanolaminates, MAX phase, Ti_3_AlC_2_, Microstructure, Oxidation behavior

## Abstract

The oxidation behavior of Ti_3_AlC_2_-based materials had been investigated at 600 °C in static air for 1000 h. It was shown that the intense increase of weight gain per unit surface area for sintered material with porosity of 22 % attributed to oxidation of the outer surface of the specimen and surfaces of pores in the bulk material. The oxidation kinetics of the hot-pressed Ti_3_AlC_2_-based material with 1 % porosity remarkably increased for the first 15 h and then slowly decreased. The weight gain per unit surface area for this material was 1.0 mg/cm^2^ after exposition for 1000 h. The intense initial oxidation of Ti_3_AlC_2_-based materials can be eliminated by pre-oxidation treatment at 1200 °C in air for 2 h. As a result, the weight gain per unit surface area for the pre-oxidized material did not exceed 0.11 mg/cm^2^ after 1000 h of exposition at 600 °C in air. It was demonstrated that the oxidation resistance of Ti_3_AlC_2_-based materials can be significantly improved by niobium addition.

## Introduction

Recently, new classes of materials based on layered carbide Ti_3_AlC_2_ have attracted great attention of material scientists due to their exceptional properties. This carbide belongs to the so-called MAX phases which have a chemical formula M_*n* + 1_AX_*n*_—where M is an early transition metal, A is an A-group element, and X is carbon and/or nitrogen. The crystal structure of MAX phases can be described as octahedral ternary metal carbide and/or nitride sandwiched by close-packed layers of A-element. These materials have good thermal and electrical conductivity, low density, high strength and Young’s modulus, excellent thermal shock resistance, high chemical resistance, relatively low thermal expansion coefficient, and good machinability [1–3]. Owing to such combination of properties, they have been suggested for various applications, especially as high-temperature structural materials. This requires comprehensive investigations of oxidation resistance of Ti_3_AlC_2_-based materials. Barsoum et al. [4] had demonstrated that for Ti_n+1_AlX_n_ compounds oxidized in the 800–1000 °C temperature range, the scale composed mainly of rutile-based solid solution (Ti_1 − y_Al_*y*_)O_2 − *y*/2_, where *y* < 0.05 and some Al_2_O_3_. The oxidation process occurred by the inward diffusion of oxygen and the outward diffusion of Al, Ti, C, and N. It was revealed that the formation of a thin layer of Al_2_O_3_ preceded the nucleation and growth of TiO_2_ at the early stages of oxidation [5]. The scale formed at higher temperatures consisted of a continuous Al_2_O_3_ inner layer and outer layer, changed from rutile TiO_2_ at temperatures below 1200 °C to a mixture of Al_2_TiO_5_ and TiO_2_ at 1300 °C [6]. Taotao [7] had reported that the scale of an un-dense Ti_3_AlC_2_ material containing 3 wt. % TiC oxidized at 1000 °C in air consisted of three layers, including an outer un-dense TiO_2_ layer adhering to a little Al_2_O_3_, a thick intermediate TiO_2_ + Al_2_O_3_ mixed layer, and a thin inner Al_2_O_3_ layer with some pores.

In spite of thorough research of oxidation behavior of Ti_3_AlC_2_-based materials at high temperature, only a few results obtained at intermediate temperatures have been reported [8, 9]. Taking into account the anomalously intense oxidation of Ti_3_AlC_2_-based material at 500 °C and especially at 600 °C [8], the investigation of oxidation behavior of these materials at intermediate temperatures has a great importance.

In this work, the Ti_3_AlC_2_-based materials have been oxidized at 600 °C in air for 1000 h. The influence of porosity, pre-oxidation treatment, and niobium addition on the oxidation resistance of these materials has been investigated.

## Review

### Experimental

**Fig. 1:**
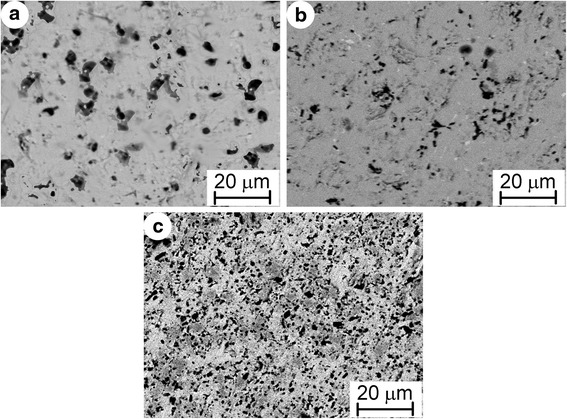
Back-scattered electron images of the polished surfaces: **a** Ti_3_AlC_2_-based material with 22 % porosity, **b** Ti_3_AlC_2_-based material with 1 % porosity, and **c** Nb alloyed Ti_3_AlC_2_-based material with 1 % porosity

**Fig. 2:**
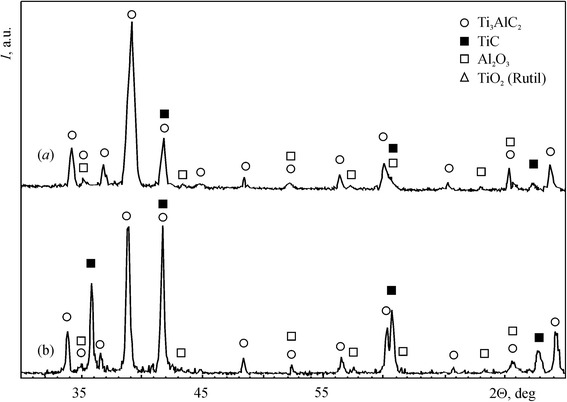
XRD patterns obtained from the surface of the Ti_3_AlC_2_-based material with 1 % porosity (**a**) and Nb alloyed Ti_3_AlC_2_-based material with 1 % porosity (**b**)

The materials used in this work were initially sintered in vacuum from mixture of TiC, TiH_2_, and Al powders and then hot pressed at 1350 °C under pressure of 30 MPa for 1 h. The phase composition of a sintered material consisted of 95 wt. % Ti_3_AlC_2_ and 5 wt. % TiC. Figure 1a shows the back-scattered electron image of a polished surface of this material. The equiaxed grains of Ti_3_AlC_2_, fine particles of TiC, and big pores were observed. The porosity of the material was 22 %. After hot pressing, refinement of the structure and reduction of the porosity to 1 % occurred (Fig. 1b). The amount of Ti_3_AlC_2_ was decreased to 92 wt. % and TiC increased to 6 wt. % (Fig. 2a). Additionally, 2 wt. % Al_2_O_3_ was revealed in this material. The material alloyed with 3.5 wt. % Nb fabricated by the same process as previous material consisted of 56 wt. % (Ti, Nb)_3_AlC_2_, 41 wt. % TiC, and 3 wt. % Al_2_O_3_ (Fig. 2b). The structure of this material includes equiaxed grains of Ti_3_AlC_2_ and TiC and uniformly distributed fine particles of Al_2_O_3_ and small pores (Fig. 1c).

The isothermal oxidation tests were carried out at the temperature of 600 °C in static air using tree rectangular bars with dimensions of 20 × 5 × 3 mm for each material. The specimens were cut by the electrical discharge method, abraded to 1000 grit with SiC paper, and polished by diamond past. The oxidation tests were divided into five stages which had the duration: first stage, 15 h; second stage, 245 h; and the last three stages, 250 h. Each stage consisted of heating to 600 °C in air, exposition during determined time, and cooling to room temperature. The weight of the specimens was measured before the test and after each stage by analytical balance. The accuracy of the weight measuring was ±10^−4^ g. The oxidation resistance of materials tested was characterized by weight gain per unit surface area ΔW/S. The phase composition of the materials was analyzed by X-ray diffractometry (Dron-3M, Russia). Diffraction data were processed by the Rietveld method using the PowderCell program. Scanning electron microscopy (EVO 40 XVP (Carl Zeiss, Germany)) coupled with energy dispersive spectroscopy (EDS) (INCA ENERGY 350 (Oxford Instruments, UK)) was used to study the structure and quantitative elemental content of the bulk material and the oxidized scale.

## Results and Discussion

**Fig. 3:**
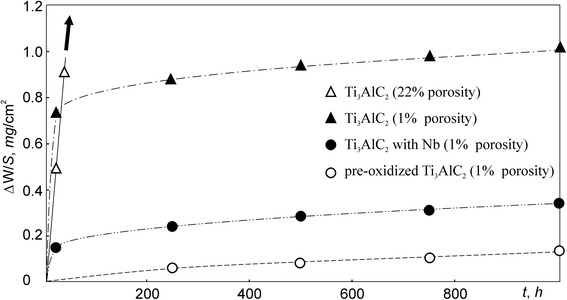
Oxidation kinetics for the Ti_3_AlC_2_-based materials at 600 °C in air

**Fig. 4:**
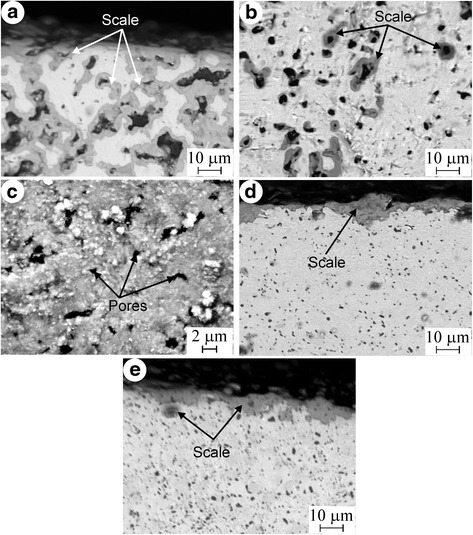
Back-scattered electron images of the scales formed at 600 °C for 1000 h: **a** Ti_3_AlC_2_-based material with 22 % porosity, **b** oxidized pores of Ti_3_AlC_2_-based material with 22 % porosity, **d** Ti_3_AlC_2_-based material with 1 % porosity, **e** Nb alloyed Ti_3_AlC_2_-based material with 1 % porosity, and **c** scale surface of Ti_3_AlC_2_-based material with 1 % porosity after 15 h oxidation

The dependences of weight gain per unit surface area on oxidation time at 600 °C for Ti_3_AlC_2_-based materials tested are presented in Fig. 3. It can be seen that the ΔW/S for material with porosity of 22 % monotonically increases and reaches the value of 24 mg/cm^2^ after exposition of 437 h. Because of the very high value of the ΔW/S, the test for this material had been stopped. The rapid oxidation of the Ti_3_AlC_2_-based material with porosity of 22 % can be explained by intense penetration of oxygen into the material through the pores. As a result, not only outer surface of the specimen but also the surfaces of pores in the bulk material were oxidized (Fig. 4a, b).

The oxidation kinetics for the Ti_3_AlC_2_-based material with 1 % porosity at 600 °C remarkably increases on the first stage of the test (Fig. 3). After 15 h, the weight gain per unit surface area increases more slowly, and after 1000 h, exposition is 1.0 mg/cm^2^. Table 1 presents an average quantity of chemical elements in bulk material and scale, obtained by the EDS method. It can be seen that carbon misses instead of oxygen were present in the scale. This means that the surfaces of Ti_3_AlC_2_ and TiC particles completely oxidized to TiO_2_, Al_2_O_3_, and gaseous CO or CO_2_ phases which volatilized from the scale. The volatilization of gaseous phases leads to pore formation in the oxide layer (Fig. 4c). The overall equations for the oxidation reactions are:

$$ 4\mathrm{T}{\mathrm{i}}_3\mathrm{A}\mathrm{l}{\mathrm{C}}_2+23{\mathrm{O}}_2=12\mathrm{T}\mathrm{i}{\mathrm{O}}_2+2\mathrm{A}{\mathrm{l}}_2{\mathrm{O}}_3+8\mathrm{C}{\mathrm{O}}_2\left(\mathrm{g}\right),\mathrm{TiC}+2{\mathrm{O}}_2=\mathrm{T}\mathrm{i}{\mathrm{O}}_2+\mathrm{C}{\mathrm{O}}_2\left(\mathrm{g}\right). $$

**Table 1:** EDS analysis results of the Ti_3_AlC_2_-based materials with 1 % porosity (at. %)

Materials	Ti		Al		C		Nb		O	
	Bulk	Scale	Bulk	Scale	Bulk	Scale	Bulk	Scale	Bulk	Scale
Ti_3_AlC_2_	51.3	23.7	14.8	8.1	34.6	0	0	0	0	68.2
Ti_3_AlC_2_ with Nb	44.1	25.3	14.9	3.3	42.8	0	1.4	0.6	0	70.1

Wang and Zhou [8] had demonstrated that the scale formed at 600 °C on the surface of a Ti_3_AlC_2_ material with 5 val. % TiC consisted of amorphous Al_2_O_3_, anatase, and rutile TiO_2_. The formation of anatase from Ti_3_AlC_2_ led to the increase of stress due to the difference of their volume expansion. Therefore, the rapid increase of ΔW/S value for the Ti_3_AlC_2_-based material with 1 % porosity on the first stage of the test can be associated with intense scale formation as well as with low protective property of the thin scale due to microcracks and also with penetration of oxygen through micropores into the bulk material (Fig. 4c). After long-term exposition, when micropores were covered with oxides and the scale thickness was increased (Fig. 4d), the inward diffusion of oxygen and outward diffusion of Ti and Al became slow. As a result, the oxidation kinetics was decreased. Based on these results, it can be assumed that the preliminary oxidation to form the protective layer of oxides would increase the oxidation resistance of Ti_3_AlC_2_-based materials. The pre-oxidation at 1000–1300 °C for 2 h provides the formation of a dense scale which consists of Al_2_O_3_ and rutile TiO_2_ without anatase TiO_2_ [10]. In the present study, the pre-oxidation of the Ti_3_AlC_2_-based material with 1 % porosity was performed at 1200 °C in air for 2 h. The weight gain per unit surface area after pre-oxidation is 1.7 mg/cm^2^_._ The long-term oxidation resistance was investigated at 600 °C in air for 1000 h. As can be seen in Fig. 3, the pre-oxidized material demonstrates the negligible increase of weight gain per unit surface area during all tests. The value of ΔW/S for this material not exceeds a 0.11 mg/cm^2^ after a 1000-h exposition.

**Fig. 5:**
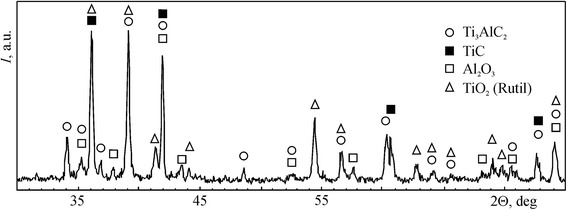
XRD pattern obtained from the scale surface of the Nb alloyed Ti_3_AlC_2_-based material with 1 % porosity after oxidation at 600 °C in air for 1000 h

The influence of Nb on the oxidation resistance of a Ti_3_AlC_2_-based material with 1 % porosity had been investigated. The cross-sectional view of the oxidized surface is shown in Fig. 4e. Despite a greater content of TiC, the weight gain per unit surface area for this material increases more slowly as compared with material without Nb (Fig. 3). The ΔW/S is 0.34 mg/cm^2^ after exposition at 600 °C for 1000 h. This value is approximately three times smaller than that for Ti_3_AlC_2_-based materials without Nb. Barsoum et al. [11] had shown that TiC has a deleterious effect on the oxidation kinetics of Ti_3_SiC_2_-based materials which also belong to isotypic structure M_*n* + 1_AX_*n*_. The contradiction of this conclusion with the results obtained in present work can be explained by a positive effect of Nb on the oxidation resistance of Ti_3_AlC_2_-based materials. The EDS analysis shows that the content of Ti, Al, and Nb in the scale of the Ti_3_AlC_2_-based material with Nb is less than that in the bulk material (Table 1). On the other hand, the content of Al in the scale of this material is significantly less than that of the material without Nb. This means that Nb impedes the outward diffusion of Al. As the result, the formation of Al_2_O_3_ is restricted. The XRD analysis shows that the scale of the Ti_3_AlC_2_-based material with Nb consists mainly of TiO_2_ and minor quantity of Al_2_O_3_ (Fig. 5). According to [6], the formation of a dense Al_2_O_3_ layer is responsible for high oxidation resistance of Ti_3_AlC_2_-based materials. Thus, a material with Nb ought to be less oxidation resistant than a material without Nb. Jiang et al. [12] had concluded that Nb in solid solution with TiO_2_ impedes mass transfer in TiO_2_ and improves the oxidation resistance of Ti–Al–Nb alloys greater than Al. Evidently, in case of Ti_3_AlC_2_-based materials, the positive effect of Nb manifests in the same manner.

## Conclusions

The investigation of oxidation resistance of Ti_3_AlC_2_-based materials had been carried out at 600 °C in static air for 1000 h. The results showed that the weight gain per unit surface area for sintered Ti_3_AlC_2_-based materials with porosity of 22 % monolithically increased and after 437 h reached the value of 24 mg/cm^2^. The drastic increase of oxidation kinetics of this material caused by intense oxidation not only the outer surface of specimen but also the surfaces of pores. The weight gain per unit surface area for hot-pressed Ti_3_AlC_2_-based materials with 1 % porosity intensively increased for the first 15 h of oxidation, and then the oxidation kinetics slowly decreased. The pre-oxidation at 1200 °C for 2 h eliminated the initial oxidation of this material at 600 °C. It was revealed that the niobium addition significantly improves the oxidation resistance of the Ti_3_AlC_2_-based material.
